# Factors affecting lung cancer stage at diagnosis and receipt of surgery in Ontario: A population-based analysis

**DOI:** 10.1371/journal.pone.0354330

**Published:** 2026-07-28

**Authors:** Nader M. Hanna, Saad Shakeel, Gileh-Gol Akhtar-Danesh, Noori Akhtar-Danesh, Christian Finley

**Affiliations:** 1 Department of Surgery, McMaster University, Hamilton, Ontario, Canada; 2 Department of Surgery, Southlake Health, Newmarket, Ontario, Canada; 3 Department of Surgery, University of Western Ontario, London, Ontario, Canada; 4 Department of Surgery, University of Manitoba, Winnipeg, Manitoba, Canada; 5 School of Nursing, McMaster University, Hamilton, Ontario, Canada; University of Illinois Urbana-Champaign College of Veterinary Medicine, UNITED STATES OF AMERICA

## Abstract

Lung cancer is the leading cause of cancer mortality in Canada, with most cases diagnosed at advanced stage. Despite universal healthcare, social determinants of health may impact stage at diagnosis and access to treatment. We examined the association between these factors, stage at diagnosis, and receipt of surgery in Ontario. We conducted a population-based retrospective cohort study of adults diagnosed with lung cancer in Ontario from 2007–2023 using linked administrative health data. Stage was obtained from the Ontario Cancer Registry. Factors included income, primary care attachment, immigration status, geographic region, and distance to the nearest cancer centre. Logistic regression identified factors associated with stage IV diagnosis. Among 106,867 patients with available stage data, 52.6% were diagnosed with stage IV disease; 19.1% were stage I. Patients without a family physician were significantly more likely to present with stage IV cancer compared with those fully rostered (62.1% vs 51.5%; adjusted OR 1.55, p < 0.001). Females had lower likelihood of stage IV diagnosis than males (OR 0.85, p < 0.001). Increasing household income was associated with reduced likelihood of stage IV disease (OR 0.92 for highest vs lowest quintile, p < 0.001). Immigrants were less likely to be diagnosed with stage IV cancer than non-immigrants (OR 0.77, p = 0.001). Frailty was associated with higher odds of stage IV disease (OR 1.01, p = 0.009). Surgical resection was performed in 56.1% of patients with stage I disease and 1.4% with stage IV disease. Receipt of surgery decreased with advancing age (27.0% < 50 years vs 7.3% ≥ 80 years), higher comorbidity burden (25.4% with 0 vs 5.0% with ≥5 comorbidities), and lack of primary care attachment. Marked social inequities in lung cancer stage at diagnosis and surgical treatment persist in Ontario. Interventions addressing primary care attachment and upstream social determinants may improve early detection and access to curative therapy.

## Introduction

Lung cancer remains the leading cause of cancer mortality in Canada, with nearly 30,000 new cases diagnosed annually and more deaths than any other malignancy [1,2]. Approximately half of all lung cancer cases in Canada are diagnosed at stage IV, when treatment options are most limited and survival is poor. Early-stage detection is critical, as five-year survival for stage I lung cancer is reported at 61%, compared with approximately 3% for stage IV disease [3].

Despite its public health importance, lung cancer continues to be diagnosed late in many populations [4]. Social determinants of health (SDOH), such as socioeconomic status (SES), continuity of primary care, immigration status, and geographic access to services, are increasingly recognized as factors influencing both stage at diagnosis and subsequent treatment patterns [5–7]. SES inequalities in lung cancer outcomes have been documented in multiple settings, with lower income and reduced health care access linked to more advanced disease and poorer survival [8]. Additionally, migrant populations comprise a significant proportion of Canada’s demographic profile, yet evidence is mixed on whether immigrant status independently influences stage at diagnosis or outcomes. Population studies in Ontario suggest that immigrant status may not be associated with a higher likelihood of late-stage diagnosis, but variability persists by sociodemographic context [9,10].

Although Canadians benefit from a universal healthcare system, social disparities influence access to care, treatment received, and cancer outcomes [11]. In this study, we use population-level health administrative databases to identify associations between SDOH influence lung cancer stage at diagnosis and subsequent treatment.

## Methods

### Setting, data platform, and ethics

This analysis was conducted in Ontario, Canada, using linked population-level administrative health data held at the Institute for Clinical Evaluative Sciences (ICES). Datasets were connected using unique, encoded identifiers and were analyzed within the ICES secure environment. ICES is an independent, non-profit research institute; under Ontario’s health information privacy legislation, it is authorized to collect and use health care and demographic information without individual consent for health system evaluation and improvement. Ontario is the most populous Canadian province, with more than 16 million residents. This study follows the same overall methodological approach and data definitions as our previously published work [11]. The Hamilton Integrated Research Ethics Board (REB) approved this study (HiREB #17274). The REB waived the need for patient consent as the data were fully anonymized prior to access. Data were accessed for research purposes on 6^th^ January 2025.

### Cohort creation

We created a retrospective cohort from the Ontario Cancer Registry (OCR) that included adults diagnosed with lung cancer from January 2007 to December 2023. Lung cancer cases were identified using ICD‑O topography codes C34.0, C34.1, C34.2, C34.3, C34.8, and C34.9 (malignant neoplasms of the bronchus and lung, including main bronchus; upper, middle, and lower lobes; overlapping lesions; and unspecified sites). We included individuals with all stages using the OCR *best stage* variable, which prioritizes pathological staging when available. We then categorized patients by whether or not they received surgery. Surgical procedures were captured using the Canadian Classification for Health Interventions (CCI). To minimize inclusion of prevalent malignancies and subsequent competing cancer events, we excluded individuals with any cancer diagnosis in the five years prior to the index lung cancer diagnosis and those with another cancer diagnosis in the five years following the index diagnosis.

### Data sources

The OCR captures demographic characteristics, diagnostic information, and deaths for more than 98% of incident cancers in Ontario [12]. The OCR was linked to other administrative data holdings to define treatment exposure, covariates, and outcomes. Surgical interventions were derived from CCI. Additional covariates were obtained from the Registered Persons Database (RPDB), Ontario Health Insurance Plan (OHIP) claims, Same Day Surgery (SDS), Discharge Abstract Database (DAD), Local Health Integration Network (LHIN) files, Postal Code Conversion File (PCCF), Ontario Marginalization Database (ONMARG), National Ambulatory Care Reporting System (NACRS), and the IRCC Permanent Residents database (CIC).

### Outcome

The primary outcome was stage of cancer at diagnosis. The secondary outcome was receipt of surgery.

### Covariates

We examined age, sex, and year of diagnosis (all categorized), along with comorbidity and frailty measures derived using the Johns Hopkins ACG System [13]. Comorbidity was calculated from OHIP and DAD records in the 6–30 months preceding the diagnosis date and categorized per ACG definitions; frailty was derived using the same system and analyzed in categorized form. Geographic variation was assessed using Ontario’s 14 LHINs, grouped into four regions: Central, Southwest, East, and North. Social determinants of health included household income, neighbourhood income, family physician status, immigration status, and distance to the nearest regional cancer centre. Individuals who immigrated to Canada ≤5 years before diagnosis were classified as new/recent immigrants.

### Statistical analysis

We described the cohort using descriptive statistics and assessed associations between individual stage and each covariate using chi-square tests. Logistic regression was used to identify factors associated with a Stage IV diagnosis. Analyses were conducted using Stata/MP 15.1 (Stata Corporation, College Station, TX, USA).

## Results

We identified 130,825 patients diagnosed with lung cancer between January 2007 and December 2023. Of these, 106,867 (81.7%) had stage data available. Males comprised 50.6% (54,124 / 106,867) of the total cohort. The largest age group were those between 70–79 years (33.8%) with the remaining aged <50 years (2.9%), 60–69 years (30.0%), and ≥80 (20.2%). Stage distributions were as follows: Stage I 20,393 (19.1%); Stage II 7,826 (7.3%); Stage III 22,427 (21.0%); Stage IV 56,221 (52.6%). In the period 2020–2023 there were more Stage I and fewer Stage IV diagnoses than previous years. Table 1 illustrates the demographic factors associated with stage at diagnosis.

**Table 1:** Demographic factors associated with clinical stage at presentation.

	Stage I	Stage II	Stage III	Stage IV	Total	p-value
**Age groups** <50 50-59 60-69 70-79 >80	539 (17.5) 2126 (15.1) 5794 (18.1) 7540 (20.9) 4394 (20.3)	181 (5.9) 890 (6.4) 2308 (7.2) 2782 (7.7) 1665 (7.7)	583 (19.0) 3041 (21.7) 6855 (21.4) 7725 (21.3) 4223 (19.6)	1770 (57.6) 7964 (56.8) 17062 (53.3) 18124 (50.1) 11301 (52.3)	3073 14021 32019 36171 21583	<0.001
**Sex** Male Female	8802 (16.2) 11591 (21.9)	4042 (7.5) 3783 (7.2)	11698 (21.6) 10728 (20.3)	29582 (54.6) 26639 (50.5)	54124 52741	<0.001
**Comorbidities** 0 1-2 3-4 >5	6241 (24.5) 4628 (20.8) 1327 (23.4) 404 (5.9)	2165 (8.5) 2025 (9.1) 552 (9.7) 206 (3.0)	4888 (19.2) 5470 (24.6) 1400 (24.7) 2552 (37.5)	12167 (47.8) 10041 (45.3) 2386 (42.1) 3639 (53.5)	25461 22164 5665 6801	<0.001
**Frailty** No Yes	18113 (18.9) 2280 (20.7)	7009 (7.3) 817 (7.4)	20381 (21.3) 2046 (18.6)	50334 (52.5) 5887 (53.4)	95837 11030	<0.001
**Year** 2007-2009 2010-2014 2015-2019 2020-2023	2972 (17.1) 5927 (16.5) 7460 (21.3) 4034 (21.4)	956 (5.5) 2759 (7.7) 2696 (7.7) 1415 (7.5)	4726 (27.3) 6970 (19.4) 6827 (19.6) 3904 (20.7)	8692 (50.1) 20132 (56.3) 17910 (51.3) 9487 (50.3)	17346 35788 34893 18840	<0.001

Table 2 depicts the social determinants of health associated with stage at diagnosis. There were a greater number of patients not rostered with a family physician diagnosed with stage IV lung cancer (62.1%) compared with those fully rostered (51.5%) and those only virtually rostered (55.7%). Those deemed to be immigrants were more likely to be diagnosed as stage IV (55.6%) compared to non-immigrants (52.4%). New immigrants (those living in Canada ≤5 years before diagnosis) were more likely to be diagnosed as stage I (22.0% vs 19.0%) and less likely to be diagnosed as stage IV (49.7% vs 52.6%) compared to non-immigrants. There was a similar distribution of stage amongst the household income quintiles and neighbourhood income quintiles. Patients living within 50 km of a regional cancer centre were more likely to be diagnosed at stage IV compared to those living > 100 km from the nearest cancer centre (53.4% vs 49.8%). Patients living in the southwest region of the province were the least likely to be diagnosed at stage I (16.9%) whereas those in the central region of the province were the most likely to be diagnosed with stage IV disease (52.6%).

**Table 2:** Social determinants of health associated with clinical stage at presentation.

	Stage I	Stage II	Stage III	Stage IV	Total	p-value
**Family Physician** Rostered Virtually rostered Not rostered	17244 (19.8) 2636 (16.5) 513 (12.2)	6489 (7.4) 1084 (6.8) 253 (6.1)	18255 (21.1) 3354 (21.0) 818 (19.6)	44734 (51.5) 8892 (55.7) 2595 (62.1)	86722 15966 4179	<0.001
**Immigration** No Yes	19025 (19.0) 1368 (19.8)	7403 (7.4) 423 (6.1)	21145 (21.2) 1282 (18.5)	52380 (52.4) 3841 (55.6)	99953 6914	<0.001
**New Immigrant** No Yes	20234 (19.0) 159 (22.0)	7771 (7.3) 55 (7.6)	22278 (21.0) 149 (20.6)	55862 (52.6) 359 (49.7)	106145 722	0.206
**Household Income** Quintile 1 Quintile 2 Quintile 3 Quintile 4 Quintile 5	2358 (18.6) 3342 (19.1) 3995 (19.2) 4383 (18.4) 6127 (19.7)	834 (6.6) 1338 (7.6) 1514 (7.3) 1720 (7.2) 2327 (7.4)	2519 (20.0) 3663 (20.9) 4411 (21.2) 5254 (22.1) 6353 (20.4)	6913 (54.7) 9131 (52.3) 10896 (52.3) 12392 (52.1) 16310 (52.4)	12624 17474 20816 23749 31117	<0.001
**Neighbourhood Income** Quintile 1 Quintile 2 Quintile 3 Quintile 4 Quintile 5	5009 (18.7) 4524 (18.7) 3877 (19.0) 3588 (19.2) 3310 (20.2)	1999 (7.5) 1745 (7.2) 1463 (7.1) 1376 (7.4) 1196 (7.3)	5750 (21.4) 5114 (21.2) 4302 (21.1) 3912 (20.9) 3268 (20.0)	14078 (52.4) 12708 (52.8) 10727 (52.6) 9838 (52.6) 8603 (52.5)	26836 24091 20369 18714 16377	0.006
**Distance** <50km 50-100 km >100km	15292 (19.1) 3613 (19.3) 1446 (18.2)	5724 (7.2) 1439 (7.7) 642 (8.1)	16264 (20.3) 4232 (22.6) 1886 (23.8)	42725 (53.4) 9410 (50.3) 3955 (49.8)	80005 18694 7929	<0.001
**Regions** Central Southwest Eastern Northern	5431 (20.5) 6034 (16.9) 6128 (20.4) 2774 (19.0)	1818 (6.9) 2588 (7.3) 2224 (7.4) 1178 (8.0)	4808 (18.1) 7755 (21.8) 6555 (21.9) 3280 (22.5)	14500 (54.6) 19209 (53.9) 15088 (50.3) 7339 (50.4)	26557 35586 29995 14571	<0.001

Table 3 demonstrates factors associated with a diagnosis of stage IV lung cancer using logistic regression. Females were significantly less likely to be diagnosed with stage IV disease (OR=0.85, p-value <0.001). The likelihood of a stage IV diagnosis significantly decreased with advancing age (e.g., OR=0.82, p-value <0.001 for >80 years compared to < 50 years), increasing household income (OR=0.92, p-value <0.001 for quintile 5 compared to quintile1), immigrant status (OR=0.77, p-value 0.001), and distance from the nearest cancer centre (OR=0.90, p-value <0.001 for >50km compared to <50km). Likelihood of stage IV diagnosis increased with frailty (OR=1.01, p-value 0.009), not being rostered with a family physician (OR=1.55, p-value<0.001 when compared with those fully rostered). The likelihood of a stage IV diagnosis significantly increased over time (OR=1.30 p-value<0.001 for years 2010–2014 compared to 2007–2009) but then levelled out in the later period of years 2020–2023 (OR=1.03, p-value 0.133). Those in the east and north were significantly less likely to receive a stage IV diagnosis compared to those in the Central region (OR=0.87 p-value <0.001, OR=0.88 p-value <0.001, respectively).

**Table 3:** Regression analysis demonstrating likelihood of Stage IV diagnosis.

	OR	95%CI	p-value
**Age groups** <50 (reference) 50-59 60-69 70-79 >80	--- 0.97 0.86 0.76 0.82	--- 0.89-1.05 0.79-0.92 0.70-0.82 0.75-0.88	--- 0.479 <0.001 <0.001 <0.001
**Sex** Male (reference) Female	--- 0.85	--- 0.83-0.87	--- <0.001
**Frailty Index** No (reference) Yes	--- 1.06	--- 0.1.01-1.10	--- 0.009
**Family Physician** Rostered (reference) Virtually rostered Not rostered	--- 1.16 1.55	--- 1.12-1.20 1.45-1.66	--- <0.001 <0.001
**Immigration** No (reference) Yes	--- 0.77	--- 0.67-0.90	--- 0.001
**Household Income** Quintile 1 (reference) Quintile 2 Quintile 3 Quintile 4 Quintile 5	--- 0.93 0.95 0.94 0.92	--- 0.72-1.01 0.74-1.03 0.65-0.92 0.58-0.82	--- 0.003 0.029 0.004 <0.001
**Distance** <50km (reference) >50km	--- 0.90	--- 0.88-0.93	--- <0.001
**Year** 2007-2009 (reference) 2010-2014 2015-2019 2020-2023	--- 1.30 1.07 1.03	--- 1.25-1.34 1.03-1.11 0.99-1.08	--- <0.001 <0.001 0.133
**Region** Central (reference) Southwest Eastern Northern	--- 1.02 0.87 0.88	--- 0.98-1.05 0.84-0.90 0.85-0.92	--- 0.288 <0.001 <0.001

Table 4 illustrates factors associated with receipt of surgery for lung cancer. 56.1% of stage I patients had surgery whereas 1.4% of patients with stage IV lung cancer underwent surgical resection. The likelihood of receiving surgery decreased as age increased (27.0% of those aged <50 compared to 7.3% of those aged ≥80 years). Increased comorbidity burden was associated with less likelihood of receiving surgery (25.4% for 0 comorbidities versus 5.0% for ≥5 comorbidities). Those not rostered with a family physician were less likely to receive surgery (9.3%) compared with fully rostered (18.8%) and virtually rostered (14.9%) patients. Immigrants, including recent immigrants, were more likely to receive surgery than non-immigrants (22.0% vs 17.5%). Increasing household income was associated with a reduced likelihood of receiving surgery, whereas increasing neighbourhood income was associated with an increased likelihood of receiving surgery. Those in the central region of the province were most likely to receive surgery (21.2%) whereas those in eastern region were the least likely (15.0%).

**Table 4:** Factors associated with receipt of surgery.

	No Surgery	Surgery	Total	p-value
**Stage** I II III IV	8945 (43.9) 3603 (46.0) 19219 (85.7) 55447 (98.6)	11448 (56.1) 4223 (54.0) 3208 (14.3) 774 (1.4)	20393 7826 22427 56221	<0.001
**Age groups** <50 50-59 60-69 70-79 >80	2642 (73.0) 12498 (78.3) 29349 (78.2) 35468 (80.9) 27633 (92.7)	975 (27.0) 6470 (21.7) 8198 (21.8) 8409 (19.1) 2183 (7.3)	3617 15968 37547 43877 29816	<0.001
**Sex** Male Female	55737 (84.5) 51851 (80.0)	10238 (15.5) 12997 (20.0)	65975 64848	<0.001
**Comorbidities** 0 1-2 3-4 >5	22946 (74.6) 22132 (81.4) 6233 (86.7) 7747 (95.0)	7835 (25.4) 5058 (18.6) 958 (13.3) 405 (5.0)	30781 27190 7191 8152	<0.001
**Frailty** No Yes	92845 (80.8) 14745 (92.6)	22049 (19.2) 1186 (7.4)	114894 15931	<0.001
**Family Physician** Rostered Virtually rostered Not rostered	85594 (81.2) 17006 (85.1) 4990 (90.7)	19737 (18.8) 2984 (14.9) 514 (9.3)	105331 19990 5504	<0.001
**Immigration** No Yes	100856 (82.5) 6734 (78.0)	21333 (17.5) 1902 (22.0)	122189 8636	<0.001
**Household Income** Quintile 1 Quintile 2 Quintile 3 Quintile 4 Quintile 5	12365 (79.5) 17107 (80.4) 20750 (81.5) 24257 (83.0) 31810 (84.1)	3195 (20.5) 4170 (19.6) 4704 (18.5) 4961 (17.0) 6010 (15.9)	15560 21277 25454 29218 37820	<0.001
**Neighbourhood Income** Quintile 1 Quintile 2 Quintile 3 Quintile 4 Quintile 5	27892 (84.7) 24342 (82.8) 20277 (81.7) 18425 (80.8) 16038 (79.6)	5033 (15.3) 5074 (17.2) 4551 (18.3) 4393 (19.2) 4110 (20.4)	32925 29416 24828 22818 20148	<0.001
**Distance** <50km 50-100 km >100km	79586 (81.7) 19368 (83.9) 8294 (83.4)	17832 (18.3) 3726 (16.1) 1649 (16.6)	97418 23094 9943	<0.001
**Year** 2007-2009 2010-2014 2015-2019 2020-2023	18069 (82.7) 32946 (83.9) 32992 (81.7) 23583 (80.8)	3791 (17.3) 6341 (16.1) 7384 (18.3) 5719 (19.5)	21860 39287 40376 29302	<0.001
**Regions** Central Southwest Eastern Northern	26126 (78.8) 35179 (82.7) 31358 (85.0) 14676 (81.7)	7026 (21.2) 7369 (17.3) 5529 (15.0) 3291 (18.3)	33152 42548 36887 17967	<0.001
**New Immigrant** No Yes	106897 (82.3) 693 (77.2)	23030 (17.7) 205 (22.8)	129927 898	<0.001

## Discussion

In this population-level study of incident lung cancer cases in Ontario, we found strong and consistent associations between SDOH and stage at diagnosis, as well as subsequent receipt of surgical treatment. This present study is the first contemporary report that investigates the association between SDOH and stage of lung cancer at diagnosis in an unselected cohort of over 106,000 patients. Despite universal health insurance in Ontario, marked gradients in stage at presentation and access to potentially curative surgery persisted across socioeconomic, geographic, and primary care attachment factors. We have previously reported that immigrants, those fully rostered with a family physician, and those in the higher income quintiles were more likely to undergo surgical resection for stage I lung cancer [11]. This current study builds on that previous work and demonstrates social inequities in lung cancer stage at diagnosis.

Younger age was strongly associated with a likelihood of stage IV disease compared to all other age groups. This pattern is inconsistent with previous literature [14,15] that shows delayed diagnosis among older adults, potentially reflecting atypical symptom presentation and lower likelihood of referral for aggressive diagnostic workup. In our data, it is likely that older patients, who carry a higher comorbidity burden, have early-stage lung cancer identified during the diagnostic workup of other comorbid conditions. However, comorbidity burden was also independently associated with more advanced stage, suggesting that clinical complexity may impede timely investigation of respiratory symptoms, reinforcing concerns that medically complex patients may face diagnostic delays despite frequent health system contact [16].

One of the most striking findings of this study was the association between primary care attachment and stage at diagnosis. Patients who were not rostered to a family physician were substantially more likely to present with stage IV disease compared with those formally rostered. Even patients who were “virtually rostered” experienced worse stage distribution than those with traditional enrollment. These findings align with previous research [17–19] demonstrating that continuity of primary care facilitates earlier cancer detection through symptom recognition, referral coordination, and diagnostic follow-up. In the context of lung cancer, where symptoms are often nonspecific and may overlap with benign respiratory disease, the absence of a consistent primary care provider may lead to fragmented care and delayed escalation of investigations. Strengthening access to comprehensive primary care may represent an opportunity to expedite lung cancer diagnosis and therefore an earlier stage.

We observed clear socioeconomic gradients in stage at diagnosis across both household- and neighbourhood-level income measures, although the magnitude of effects was subtle. Patients in the lowest income quintiles were less likely to be diagnosed with stage I disease and more likely to present with advanced-stage cancer. These findings are consistent with previous literature [5,20–23] linking socioeconomic disadvantage to later-stage lung cancer diagnosis, even in countries with universal health coverage. Several mechanisms may explain these findings, including higher prevalence of smoking and occupational exposures, competing life priorities, reduced health literacy, and barriers to accessing diagnostic imaging and specialist care [8,24]. More research in this area would be a valuable addition to identify potentially modifiable factors.

Interestingly, we found that immigrant status was a protective factor against a stage IV diagnosis. This finding is consistent with the “healthy immigrant effect” observed in Canada, whereby immigrants often arrive with better baseline health and lower smoking prevalence compared with the Canadian-born population [25,26]. However, this advantage may attenuate over time, and subgroup-specific analyses by country of origin, or duration of residence, or refugee status may reveal more nuanced patterns. Nevertheless, immigrants were more likely to receive surgery after diagnosis than non-immigrants. Language barriers, cultural differences in treatment preferences, and systemic biases may influence treatment decision-making and warrant further investigation [9,27].

Geographic distance to care was modestly but consistently associated with more advanced stage at diagnosis. Patients residing more than 100 km from cancer centres had less stage I diagnoses, more stage III disease, but fewer stage IV compared with those living within 50 km. This finding aligns with prior studies demonstrating rural–urban disparities in lung cancer outcomes, driven by reduced access to diagnostic imaging, specialist services, and multidisciplinary cancer care [28–30]. Our finding of regional variation across Ontario further supports the anticipated benefit of future health system organization and service availability on patient outcomes. Patients in the Central region of Ontario had higher proportions of advanced-stage disease compared with others, whereas the Southwest region had the lowest incidence of stage I diagnoses, reflecting longstanding disparities in cancer outcomes in specific communities [6,31]. These results highlight a gap in access that may be filled by initiatives such as mobile diagnostic services and targeted screening in underserved regions, that could potentially complement existing patient transportation initiatives [32,33].

Despite accounting for stage, disparities in surgical treatment were evident across age, sex, SES, immigration status, and primary care attachment. Older adults, men, lower neighbourhood income patients, non-immigrants, and those without a rostered family physician were less likely to undergo surgery. These findings echo prior evidence of inequities in lung cancer treatment in Canada and elsewhere [11,34,35]. Whilst some differences may reflect appropriate clinical decision-making related to frailty or comorbidity, the magnitude and consistency of disparities suggest potential under-treatment of certain groups [11]. Structural factors, including referral patterns, patient–provider communication, and implicit bias, may contribute to differential access to surgical care [8,27,36].

Our findings highlight that universal healthcare alone is insufficient to eliminate inequities in lung cancer diagnosis and treatment. Interventions to improve early detection to address upstream social and structural determinants, beyond screening eligibility, are vital to address the imbalance. Expanding organised lung cancer screening programs with proactive outreach to socially disadvantaged populations may help reduce stage disparities, particularly if coupled with primary care integration and patient navigation supports. Strengthening access to longitudinal primary care may yield benefits across the cancer care continuum, from earlier stage at diagnosis to receipt of appropriate treatment.

To our knowledge, this is the first Canadian population-level analysis to examine the relationship between SDOH and lung cancer stage at diagnosis. Key strengths of this study include the use of a large, population-based cohort spanning an extended time frame and encompassing patients treated across multiple institutions and diverse geographic regions within Ontario. However, limitations include lack of individual-level smoking data and aspects of social circumstance such as transportation access and cohabitation status, which may have influenced observed outcomes. Additionally, measures of income and distance were area-based proxies and may not fully capture individual circumstances. Furthermore, we were unable to determine whether lung cancer diagnoses among immigrants occurred before or after arrival in Canada, which may have influenced patterns of health system engagement. The available data also did not permit exploration of heterogeneity within the immigrant population by factors such as immigration class, country of origin, or language proficiency, all of which may shape access to care and treatment decisions.

## Conclusions

Despite universal healthcare coverage and the initiation of lung cancer screening, there remains significant social disparity in stage of lung cancer at diagnosis. Efforts should be aimed at a more targeted approach to vulnerable populations and widening access to primary care.
